# Dissecting Airborne Allergens

**DOI:** 10.3390/jcm12185856

**Published:** 2023-09-08

**Authors:** Javier Torres-Borrego, Manuel Sánchez-Solís

**Affiliations:** 1 Pediatric Allergy and Pulmonology Unit, Reina Sofia Children’s University Hospital, Instituto Maimónides de Investigación Biomédica de Córdoba (IMIBIC), University of Cordoba, Av. Menendez Pidal sn, 14004 Cordoba, Spain; 2 Pediatric Respiratory and Cystic Fibrosis Unit, Virgen de la Arrixaca University Children’s Hospital, Biomedical Research Institute of Murcia (IMIB), University of Murcia, Avda Teniente Flomesta, 5, 30003 Murcia, Spain; msolis@um.es

**Keywords:** airborne allergens, allergic inflammation, asthma, allergic rhinoconjunctivitis, sensitization, allergy

## Abstract

Asthma is a heterogeneous and very complex group of diseases, and includes different clinical phenotypes depending on symptoms, progression, exacerbation patterns, or responses to treatment, among other characteristics. The allergic phenotype is the most frequent, especially in pediatric asthma. It is characterized by sensitization (the production of specific IgEs) to allergens and frequent comorbidity with rhinitis as well as atopic dermatitis. Given the complexity of allergic asthma, knowledge of it must be approached from different points of view: clinical, histological, physiological, epidemiological, biochemical, and immunological, among others. Since partial approaches do not allow for the understanding of this complexity, it is necessary to have multidimensional knowledge that helps in performing the optimal management of each case, avoiding a “blind men and elephant parable” approach. Allergens are antigens that trigger the production of specific IgE antibodies in susceptible individuals, who present symptoms that will depend on the type and intensity of the allergenic load as well as the tissue where the interaction occurs. Airborne allergens cause their effects in the respiratory tract and eyes, and can be indoor or outdoor, perennial, or seasonal. Although allergens such as mites, pollens, or animal dander are generally considered single particles, it is important to note that they contain different molecules which could trigger distinct specific IgE molecules in different patients. General practitioners, pediatricians, and other physicians typically diagnose and treat asthma based on clinical and pulmonary function data in their daily practice. This nonsystematic and nonexhaustive revision aims to update other topics, especially those focused on airborne allergens, helping the diagnostic and therapeutic processes of allergic asthma and rhinitis.

## 1. Introduction

Approximately 8000 to 10,000 L of air circulate daily through the respiratory tract, which is thus continuously exposed to potentially pathogenic particles and microorganisms. The immune system is responsible for distinguishing the self from the foreign, constantly detecting antigens and particles that may be harmful so that they can be effectively eliminated. This defensive process takes place by means of complex mechanisms that are perfectly coordinated in an orderly manner, in which numerous cells and biological mediators participate, triggering a controlled inflammatory response, which will end with the elimination of the threatening agent and the resolution of the inflammation.

Allergens are proteins or glycoproteins capable of inducing IgE antibody production in genetically predisposed (atopic) individuals. Allergens can enter the body from the surrounding environment via the respiratory, digestive, or transcutaneous routes. Respiratory allergens are released from volatile particles that are in suspension in the form of aerosols. From a clinical point of view, airborne allergens are responsible for allergic rhinitis, conjunctivitis, and asthma, chronic diseases that could be seasonal or perennial, affect quality of life, and generate high healthcare costs. These are multifactorial diseases, in which genetic predisposition (atopy) is modulated by exposure to allergens, infections, and environmental factors. Larger particles are retained in the nose and conjunctiva, causing rhinoconjunctivitis, while smaller particles reach more distal regions and therefore are capable of eliciting asthma symptoms. Allergens can be classified, according to their area of exposure, into indoor allergens (mites, epithelia, fungi, and pests) or outdoor allergens (pollens and fungal spores), as well as according to the time of exposure into being perennial or seasonal. Allergens are also classified as major or minor according to the frequency (greater or lesser than 50%, respectively) of recognition by sensitized patients to an allergenic source. Panallergens, on the other hand, are proteins whose structures and functions are similar in different biological species, even though they are phylogenetically separated. Due to the homology in their amino acid sequences, they are responsible for cross-reactivity in both diagnostic tests and in clinical responses.

Adaptive or antigen-specific immunity is based on the coordinated action of T and B lymphocytes in a system capable of maintaining immunological memory that allows the development of a rapid response in the case of re-exposure to a certain antigen. The basis of allergic diseases lies in an inappropriate adaptative response driven by T helper type 2 (Th2) lymphocytes against allergens, which leads to the excessive production by B lymphocytes of isotype E immunoglobulins (IgEs) specific to one or more of such allergens, triggering tissue damage.

The current understanding of the interaction between airborne allergens and human beings is very complex and contains gaps that will gradually be resolved. This nonsystematic and nonexhaustive review includes topics that can help physicians to improve their knowledge of allergy and allergens, which will benefit better diagnostic and therapeutic management of respiratory allergic diseases in clinical practice.

## 2. Atopy

The term atopy was introduced by Coca and Cooke in 1923 [1] to describe immediate cutaneous reactions produced in response to allergens in patients with asthma and/or allergic rhinitis. Atopy was later defined as the genetic predisposition of some individuals to produce specific IgE antibodies directed against environmental allergens, resulting in the tendency of atopics to develop IgE-mediated allergic diseases. This definition does not imply the presence of clinical symptoms, but only describes immunological reactivity and the risk of allergic disease.

## 3. Allergy vs. Allergic Sensitization

The term allergy was proposed in 1906 by Von Pirquet [2], alluding to the altered reaction of the immune system of some individuals to substances that are harmless to the rest of the population. The term allergy is usually synonymous with IgE-mediated immediate hypersensitivity, being the clinical expression of an atopic predisposition, and includes asthma, rhinitis, conjunctivitis, atopic dermatitis, and allergic reactions to foods, drugs, and hymenoptera venoms. Atopy and allergy are used interchangeably in research and clinical practice to refer to immunological processes related to allergic diseases, whose familial clustering suggests hereditary transmission.

It is important to distinguish between sensitization i.e., the production of specific IgE against allergens, demonstrable by a skin prick test and/or the determination of specific serum IgE, and allergy, which implies that sensitization already produces clinical symptoms. Patients with allergic sensitization do not always manifest allergic symptoms. An individual may present positive tests against a particular allergen for which he or she has never presented a clinical allergy, and, similarly, other patients resolve their symptoms spontaneously or after receiving immunotherapy. All of them can continue to present positive tests (indicating allergic sensitization), which would suggest that those individuals have acquired tolerance via immunomodulation, constituting what is called *subclinical or asymptomatic sensitization*.

## 4. Subpopulations of T Helper Lymphocytes

T lymphocytes constitute approximately 70% of circulating lymphocytes, recognizing peptides presented to them by antigen-presenting cells (APCs), such as dendritic cells and macrophages. Three subtypes of helper lymphocytes (LTh) are described, according to the cytokine profile that they secrete once activated [3,4]. These subpopulations of LTh mutually inhibit each other in a coordinated and specialized response against microorganisms.

- Th1 lymphocytes produce interleukin 2 (IL-2), interferon gamma (IFN γ), and tumor necrosis factor β (TNF-β), promoting responses against intracellular germs due to their capacity to activate macrophages and other mechanisms of cellular immunity, in addition to inducing B lymphocytes to produce complement-binding antibodies.
- Th2 produces, among other things, IL-4, IL-5, and IL-13, whose main functions are to induce IgE antibody synthesis by plasma cells and eosinophil chemotaxis, playing a key role in the response against helminths and in allergic processes.
- Th17 lymphocytes specifically secrete IL-17, coordinating the immune response differently from Th1 and Th2, with special relevance in the response against extracellularly growing bacteria, mycobacteria, and fungi, and is believed to be involved in the pathogenesis of autoimmune diseases. They have a proinflammatory effect that serves as a transition between innate and adaptive immunity. IL-17 can stimulate neutrophilic airway inflammation, induced by infections, cigarette smoke, and pollution, but not by allergens [5].

There is a hormonal, biochemical, and immunological equilibrium that changes according to the stage of pregnancy in order to achieve tolerance towards the semiallogenic fetus. From an immunological point of view, a balance between Th1, Th2, Th17, and Treg is required to avoid pregnancy losses due to immunological rejection [6]. In the implantation phase a controlled proinflammatory Th1 response to promote angiogenesis and endometrial remodeling is required [7], subsequently giving way to a predominance of Th2 immunity at the fetal–maternal interface [6,8]. Th responses in early life appear to be highly stimulus-dependent, as can be substantially shaped by pathogens, maternal antibodies, and microbial colonization [9]. Th1 cells are proinflammatory in reaction to intracellular pathogens and vaccines, whereas Th2 is generated in response to parasites, respiratory syncytial virus (RSV), and allergens, and Th17 cells protect against extracellular pathogens [9]. Immunity in the very first years of life is especially critical, and this period is considered a window of opportunity in which an individual will predominantly develop Th1 or Th2 responses depending on the stimuli received.

## 5. Immunoglobulin E

In 1966, Kimishige and Teruko Ishizaka detected and isolated an immunoglobulin in patients sensitized to ragweed pollen that was specifically bounded to the antigens of this pollen [10]. In 1967, Johansson et al. studied a patient affected by myeloma, in whom they also found an immunoglobulin with characteristics different from those previously known [11]. The equivalence of the globulin discovered simultaneously by the two groups of researchers was later proven, and after an international consensus meeting in 1968 it was named immunoglobulin E (IgE), whose two main roles are defense against parasites and participation in allergic reactions. IgE is quite cytophilic, as its half-life is 2–3 days when free in serum but longer when bound to the surfaces of mast cells and basophils, suggesting that it is intended for local rather than systemic activity. Allergens require at least two IgE binding epitopes to facilitate the cross-linking needed to activate mast cells and basophils [12].

## 6. Pathophysiology of Allergic Reactions

For an allergen to be capable of inducing sensitization via the respiratory route, it must overcome anatomic–physiological barriers (mucociliary unit, epithelia, and mucous membranes, among others) and be phagocytized via APCs. After the processing of an allergen and migration to the secondary lymphoid organs, APCs present them, bound to the major histocompatibility complex type II, to the allergen-specific memory Th lymphocytes, which induce the differentiation and clonal expansion of allergen-specific Th2 lymphocytes. These Th2 cells secrete, among other things, interleukins IL-4 and IL-13, which induce B cells to generate allergen-specific IgE, IL-5 (promotes eosinophilic inflammation), IL-9, and IL-13 (important in bronchial hyper-responsiveness and remodeling). Repeated exposure to the allergen in a susceptible individual induces sensitization, a process in which specific IgE antibodies are produced, initially without causing symptoms. Such exposure can occur through percutaneous, respiratory, or digestive routes, and it is not known why some individuals become sensitized to allergens, or why allergic patients can show different sensitization profiles to one or more protein components of a particular allergen source.

In successive re-exposures to an allergen, the inflammatory response is much more rapid as it is mediated by preformed clones of allergen-specific Th2 cells producing specific IgE. The allergen (specifically its epitopes) interacts with IgE molecules bounded to the surfaces of tissue mast cells or peripheral basophils, causing their activation and the releasing of mediators that will produce an acute allergic reaction (bronchospasm, rhinitis, and anaphylaxis). After repeated cycles of acute inflammation followed by repair, an infiltrate of resident cells (mainly neutrophils and eosinophils) is established, releasing cytokines which, by amplifying and prolonging the inflammation, result in chronic manifestations of allergic respiratory diseases, such as bronchial hyper-responsiveness or nasal congestion, caused by allergic and nonallergenic substances such as viruses, tobacco smoke, or cold air.

## 7. Airborne Allergens

Ambient air is an example of an aerosol, which is defined as a system of solid and liquid particles dissolved in a gas. One liter of air contains a dynamic distribution of millions of particles that can remain floating in both solid and liquid forms. All other environmental conditions being equal (humidity, temperature, and air speed), the duration of particles in a suspension is inversely proportional to their size.

Allergens are proteins or glycoproteins of a molecular weight between 5 and 100 kDa, which in most cases are water-soluble. Only a small percentage of all known animal and vegetable proteins are known to be allergens [13], and it has been suggested that the allergenicity of many proteins may be determined by their homology to helminth proteins that induce Th2 responses [14]. They show the characteristic of promoting the production of specific IgE antibodies (sensitization) and activating an IgE-mediated immune response in susceptible individuals, being innocuous in the majority of the population. Thus, only subjects with a personal predisposition to produce specific IgEs to allergens will react abnormally, presenting symptoms that will depend on the type and intensity of the allergen load and the specific tissue where the interaction occurs (nose, eyes, bronchial or digestive tract). Such allergenic proteins could be structural proteins or enzymatic proteins, the latter being critical in the development of allergic sensitization and appearing to be strongly associated with heightened allergenicity [15]. Mites, cockroaches, and molds possess strong proteolytic activity [16], which increases permeability and directly activates the airway epithelium, being transported through it to interact with immune cells, leading to increased inflammation [12]. Finally, carbohydrate and lipid residues associated with allergenic proteins may act as adjuvants stimulating Th2 responses.

There are several types of allergens: pneumoallergens (inhaled), trophoallergens (ingested), occupational allergens, drug allergens, and hymenoptera venoms. Airborne allergens cause their effects on the respiratory as well as ocular tracts and can be classified, according to their area of exposure, into indoor allergens (mites, epithelia, fungi, and pests) or outdoor allergens (pollens and fungal spores), as well as according to the time of exposure into being perennial or seasonal.

Although allergen sources such as mites, pollens or animal epithelia are generally considered as a whole, it is important to keep in mind that they are composed of different proteins to which patients show individual sensitization profiles. Moreover, within these proteins, only small portions, the epitopes, can bind IgE, like a key and a lock. There are also specific epitopes for other antibodies and cells. The size of pneumoallergen-carrying particles is important: those larger than 10–20 μm will be dropped in the nasal and conjunctival mucosa, producing rhinoconjunctivitis; those between 2 and 5 μm will be deposited in the tracheobronchial tree, producing asthma; and those smaller than 1 μm will reach the alveoli.

A physician who diagnoses and treats allergic respiratory diseases must have a good knowledge of the characteristics (geographical distribution, concentration, seasonality, and avoidance recommendations) of both indoor and outdoor allergens. Most respiratory allergens meet certain characteristics (Table 1) and the requirements postulated by August Thommen in 1931 (Table 2).

## 8. Major and Minor Allergens. Panallergens, Cross-Reaction

The main allergen of a substance is the one that induces a cutaneous reaction or the production of specific IgEs in more than 50% of patients sensitized to it. On the other hand, minor allergens have a recognition frequency of less than 50% of sensitized patients. This definition is not exclusive, and an allergen can behave as a minor allergen in a given population and as a major allergen in another. In general, major allergens are usually genuine, this is species-specific, while minor allergens include various panallergens. Circulating IgE in the serum of allergic individuals can recognize different allergens in a substance, which can be demonstrated through immunoblotting.

Major allergens are usually the most abundant in the allergen source and the ones that show the greatest sensitizing capacity, so they are the first to sensitize patients, later giving way to molecular spreading, in which several years after the initial sensitization patients will show an increase in the levels of specific IgEs against the “initiator” allergen and also show polysensitization to other allergenic components of the same or other allergenic sources [17,18].

Panallergens are proteins whose structures and functions are similar in different biological species, even though they are phylogenetically separated. Due to the homology in their amino acid sequences, they are responsible for cross-reactivity, both in diagnostic tests and in clinical responses. We find example of panallergens in different species of the Oleaceae family, such as olive tree (*Olea*), ash (*Fraxinus*), and privet (*Ligustrum*), which would justify cross-sensitization to olive pollen in areas where there are no olive plantations [19,20].

## 9. International Nomenclature of Allergens

More than 16,000 protein families have been described, of which only some are allergenic. Allergens are identified according to WHO/IUIS (International Union of Immunological Societies) regulations, so that for each substance the first three letters of the genus are used followed by the first letter of the species and a number according to the order of characterization. Thus, Lol p1 is the first allergen that was identified from the grass *Lolium perenne*. The Allergome platform (www.allergome.org, accessed on 24 August 2023) is a continuously updated database that collects information on all known allergen sources, currently 2554, and more than 3200 identified allergenic molecules.

Traditionally, the etiological diagnosis of a respiratory allergy has been based on clinical history and skin prick testing, using extracts containing a mix of different allergenic molecules, and on the determination of serum IgE levels specific for this mixture of allergens. These tests are very sensitive but less specific, and to determine the individual sensitization profile of a particular patient it is necessary to use molecular tools, also known as component resolved diagnosis (CRD) [21]. The component is ultimately responsible for the allergic reaction, so the diagnosis should be aimed at identifying the specific IgE responses to these individual allergenic components. Molecular allergology allows for the differentiation between genuine (species-specific) sensitization versus cross-sensitization due to panallergens, which allows for the identification of potential candidates for allergic immunotherapy (AIT). In addition, allergen microarrays with more than 100 purified allergen molecules enable simultaneous IgE measurements using only small amounts of blood.

In geographical areas with high exposure to plants and/or high biodiversity, patients can present complex sensitization profiles, with higher frequencies of polysensitization. This is due to a combination of genetic factors and a high level of pollen exposure, resulting in a different phenotypic expression of the allergic disease [22,23].

## 10. Indoor Airborne Allergens

### 10.1. Mites

Mites are microscopic arthropods that are about 300 µm long, blind, and photophobic, that exchange O_2_ and CO_2_ through their body surfaces. Like other decomposer insects, they select food that has been predecomposed by fungi. Their life cycle from an egg to an adult mite is about 30 days. Mites belong to the class Arachnida, subclass Acari, and order Astigmata. There are two groups of dust mites, house dust mites (HDMs) and storage mites (SMs), that have been identified in household environments [24]. The most relevant families for allergenic purposes are Pyroglyphidae (*Dermatophagoides pteronyssinus*, *Dermatophagoides farinae*, and *Euroglyphus mainei*), Glycyphagidae (*Glycyphagus domesticus* and *Lepidoglyphus destructor)*, Echimyopodidae (*Blomia tropicalis*), and Acaridae (*Tyrophagus putrescentiae* and *Acarus siro*). Allergens from mites have a high sensitizing capacity, constituting through their frequency and ubiquity the main inner indoor pneumoallergen.

The ideal habitat for *Dermatophagoides* is a domestic environment in areas with temperatures above 25 °C and a relative humidity higher than 75–80%, conditions that occur in homes in coastal areas. They grow in dust, accumulate in carpets, rugs, mattresses, pillows, and curtains, and feed on human flaking. In this type of environment, they reach average concentrations of 100 to 500 mites per gram of dust, where concentrations above 100 mites/g of dust are considered to initiate allergic responses in sensitized individuals. At more than 1000 m above sea level and/or in arid environments, the *Dermatophagoides* population is drastically reduced until it disappears in situations of ambient humidity below 55%, which demonstrates their low resistance to desiccation [25].

Different allergenic components of dust mite extracts could activate innate immunity through triggering pattern recognition receptors (PRRs) and then lead to allergic inflammation [24]. The most important allergens of the mites are found in both feces (each mite produces about 10–20 feces particles per day) and bodies. The diameter of fecal particles ranges from 15 to 30 microns, so large enough to remain airborne for more than a few minutes. The most important allergens (groups 1 and 2) act as proteases [26], which in addition to inducing the production of specific IgEs are able to detach elements of the respiratory epithelium, making it more permeable to allergens and other irritating particles, acting as adjuvants in Th2 allergic inflammation and increasing bronchial hyperresponsiveness. Group 10 is a tropomyosin, a panallergen that shows cross-reactivity with tropomyosins from other arthropods, like shrimp or cockroaches.

Even if the level of exposure to mites is reduced, there is still a threshold capable of producing allergic sensitization and/or symptoms. Only a drastic decrease in mite exposure can help to improve the control of asthma and rhinitis, as shown by Boner et al. [25], with Italian asthmatic children improving when staying in an allergen-free environment in the Alps. In addition to less exposure to dust mites, this mountain environment also favors lower levels of pollens and fungi, less environmental pollution, and more sun exposure, with immunomodulatory and anti-inflammatory effects [27]. Other avoidance measures are not as effective, because although they can reduce mite concentration, they do not do so to a sufficient level to have an impact on the reduction in symptoms. There is another handicap, which is that since mites are invisible to the human eye, it is difficult for patients and families to perform avoidance measures that are not noticeable in real life. The Global Initiative for Asthma (GINA) states that there is some evidence of clinical benefit with single avoidance strategies in sensitized adults, but only limited evidence for combined avoidance strategies in children, and that these measures are complicated as well as expensive, with no validated methods for identifying those who are likely to benefit [28].

### 10.2. Pet Dander

The origin of dogs dates back to 20,000–30,000 years ago. Coming from wolves (*Canis lupus lupus)*, a symbiosis was established in which both species (dogs and humans) benefited: dogs received food and shelter whilst humans received protection, and both received emotional companionship, becoming progressively a domestic animal (*Canis familiaris*). Domestic cats *(Felis domesticus)* are descended from the African wild cat (*Felis silvestris lybica)*, which was domesticated about 9000 years ago, especially by the peoples of the Near East and Egypt, in a mutually beneficial relationship where felines removed rodent pests that ruined granaries and in return obtained food and care. In recent years we have witnessed an exponential increase in the number of pets which previously lived outside the home but now stay indoors (often in bedrooms), even when the owners are not present. In the USA there are 70% of households (90.5 million families) with pets (a total of approximately 85 million cats and 78 million dogs) [29], and 90% of Americans consider them to be part of the family [30].

About 10% of the population in developed countries shows allergy to dogs and/or cats, although only 50% of them own a pet. The explanation is that pet allergens are ubiquitous, acting as hidden “mines”, due to their presence in private and public places, such as transport, leisure areas, and even hospitals. Thus, the presence of dog and cat allergens has been demonstrated in the homes [31] and automobiles [32] of those who do not have furry pets in concentrations sufficient to generate sensitization and even an allergy (established at 1 and 8 mcg/g of dust for Fel d1 as well as 2 and 10 mcg/g for Can f1, respectively). These allergens, especially Fel d1, can float in the air for several days [33], and, even after the pet is removed from the home, persist for months in the environment, making it a public health problem, because the higher the number of people with pets the greater levels of allergens transferred, increasing their load in common spaces.

In cat allergy the clear protagonist is Fel d1, since about 95% of patients are sensitized to this secretoglobine which is present in perianal and sebaceous glands, as well as in saliva. It is carried by particles of a size between 5 and 9 microns, and so can reach the distal bronchi and induce asthma alongside rhinitis symptoms. In contrast to cats, the sensitization profiles against dogs are more diverse and there is not a single predominant allergen. Can f1 and Can f2 are lipocalins that come from saliva, and act as ligands transporting pheromones, steroids, or retinol. Mammals express exogenous lipocalins (saliva, skin, and urine), whereas humans have endogenous lipocalins. Given the homology between human lipocalins and those of other mammals, the human immune system inhibits T cell responses so as not to create a conflict of autoimmunity against its own. It has been suggested that such inhibition would favor allergic B/Th2 responses in order to avoid autoimmune responses that are potentially dangerous [34]. Another major dog allergen is Can f5, androgen-regulated prostatic kallikrein, which is greatly diminished in neutered males and nonexistent in females, so that subjects monosensitized to Can f5 will only show symptoms with unneutered male dogs. It may show cross-reactivity with human prostate-specific antigen, being responsible for allergic reactions to semen in women sensitized to Can f5 [35].

Pet allergens are carried by particles present in saliva, dander, urine, and hair. These particles, depending on their weight, are deposited on the floor and furniture or remain suspended in the air. Differences have been observed in the geometric means of Can f1 levels in the hair and skin of different breeds, but there is also a large variability between individuals of the same breed [36]. These differences may be influenced, among other things, by the number of dogs, their size, the cleanliness of a home, age, hydration, and frequency as well as type of pet washing. No differences have been observed in the levels of Can f1 in the dust in suspension in homes, which are those that really impact the respiratory system, regardless of the breed of dog. Therefore, there is no evidence to confirm that there are hypoallergenic breeds [37].

Sensitization to pet allergens is bell-shaped, i.e., at both low and high levels of exposure there is a lower frequency of sensitization, while at medium levels of exposure this frequency is higher [38]. On the other hand, immunological tolerance (understood as levels of specific protective IgG) is acquired at high levels of exposure [38]. This fact translates clinically into subjects with a high level of allergen exposure at home who acquire tolerance and barely show symptoms as long as they maintain permanent contact with their own pet, but who may lose it in case of the absence of it. This is what happens in the so-called “thanksgiving” phenomenon, whereby students who have been away from home for a long time, without contact with pets, return home and show symptoms again. Conversely, asthmatic children due to cat allergy who are on vacation at home, when returning to classrooms with a high percentage of cat owners, show more symptoms and the use of medication [39]. In this context, an elegant Swedish study showed that when pupils changed clothes when entering school, the concentration of allergens on the walls and air in the classroom, as well as on pupils’ clothes, was similar to that in other classrooms where all pupils had been banned from having pets in their homes; therefore, this may be a measure to consider [40].

A consensus of Spanish allergists [41] established recommendations on the diagnostic and therapeutic management of dog and cat allergy, among which there are avoidance measures (Table 3). Separately such measures do not have sufficient evidence of efficacy, so they should be applied in combination in order to reduce allergen exposure.

### 10.3. Cockroaches

Cockroach allergy is an important risk factor for hospital admissions, emergency department visits, and asthma morbidity, exerting a greater impact on the latter than dust mites or pets [43]. The prevalence of cockroach allergy ranges in the United States from 17 to 41%, while in Europe 25% of asthmatic children are sensitized to cockroaches [44]. Allergens from cockroaches are detected in 85% of homes in inner cities in the United States, where 60–80% of asthmatic children show a positive prick to cockroach extract [45].

Of the approximately 4000 species of cockroaches, the most frequently encountered in domestic environments and therefore of allergenic interest are *Blatella germanica*, *Periplaneta americana*, and *Blatta orientalis*. They are insects that live in humid, dark, and temperate areas and feed on plants, paper, tissues, insect remains, and food. Their allergens come from dried remains of the cytoskeleton, secretions, eggs, and fecal matter, which can become airborne and cause perennial allergic symptoms. After extermination, allergens can remain in the environment for several months, carried in particles of a size of <10 µm, with the capacity to penetrate the respiratory tract. The main allergens [46] are as follows: Bla g1 (a tropomyosin that sensitizes 30–50% of all patients), Bla g2 (60–80%), Bla g4 (60%), and Bla g5 (70%).

They produce perennial asthma, which worsens in winter, usually without clear exposure to cockroaches. It is an allergenic source that usually goes unnoticed because it is not usually part of the battery of extracts used in diagnosis via a prick test. Thus, patients can remain undiagnosed for some time, so its detection will depend on the level of suspicion of the physician who requests a prick test and/or specific IgEs, even in the absence of visible pests in the patient’s home.

Mite and cockroach proteases break the bronchial epithelial barrier, facilitating allergen entry and sensitization. These allergens can directly activate bronchial epithelial cells, inducing the production of alarmin cytokines (TSLP, IL-25, and IL-33) and chemokines with the ability to recruit inflammatory cells. IL-33 is a member of the Il-1 superfamily, located in the nucleus of epithelial and endothelial cells. Its role as an alarmin is critical in the initiation of the immune response after detecting tissue damage; after binding to its receptor ST2 activates Th2, mast cell, basophil, and eosinophil immune responses [47], making both IL-33 and ST2 therapeutic targets in asthma. In addition, most of these allergens contain glycans, which enable them to activate mast cell-bounded IgE responses.

## 11. Outdoor Airborne Allergens

### 11.1. Pollens

Pollens are the viable male gametes, essential for the reproduction of most plants. Their function is to reach, through a process called pollination, the female part of a flower of the same species and fertilize it. Pollination can be entomophilous, carried out by insects, or anemophilous, with the wind being responsible for disseminating the pollen grains through the atmosphere. Anemophilous pollens are considered the most relevant allergens in the group of respiratory allergies, and in the specific case of asthma they occupy the second place after mites.

Their most important characteristic is seasonality, so that the atmospheric concentration of a pollen occurs in certain months of the year, usually during spring and summer, depending on geographical and weather conditions. Precipitation during autumn and winter and the heat during the preceding months influence the germination and growth of plants, and therefore the amount of pollens released. The main anemophilous plant species are grasses, trees, and weeds, whose pollen covering (exine) provides them with resistance to adverse environmental conditions. *Poacea* (grasses) are the most important family causing pollen allergy worldwide. The most relevant trees are those of the *Fagaceae* order (birch, hazel, and oak), predominant in Northern Europe, while in Mediterranean Europe olive pollen (*Olea*) is of special interest. The best-known weeds are *Parietaria* and *Artemisia*, both in Europe, and *Ambrosia* (ragweed), in North America. For practical purposes, physicians should be aware of the pollination periods of the different allergenic plants in the geographic areas where they practice, as exposure to pollen peaks increases the risk of allergic and asthmatic symptoms [48]. As different allergenic sources pollinate simultaneously, it can be difficult to identify the cause of a patient’s symptoms.

Airborne pollen concentrations at ground level are higher on windy and sunny days, as well as following a circadian rhythm, being highest in the morning, during pollen emission, and in the evening, as less warm air descends from the upper layers of the atmosphere. The pollen calendar consists of a graphic representation that summarizes the annual distribution of the main types of pollen prevalent in a specific geographical location, data that are of special relevance for patients and physicians when establishing diagnostic, therapeutic, and preventive strategies. Pollen grains, with diameters ranging from 10 to 100 µm, are retained in the conjunctiva and upper respiratory tract, mainly causing rhinoconjunctivitis.

Pollen is a complex mixture of allergenic proteins and other nonallergenic products [49]. Some of these substances are lipids that exhibit strong cross-reactivity with leukotriene B4 and prostaglandin E2; such eicosanoids act as allergen-independent proinflammatory factors that contribute to allergic inflammation [50]. Moreover, their enzymes can produce reactive oxygen species [51]. Grains of pollen are too large to access the bronchi, except when exposed to an osmotic rupture or environmental degradation [50], which release submicronic respirable allergens that are integrated into particles smaller than 5–8 µm, becoming bioaerosols with a significant allergenic load [52]. Such subpollen particles remain airborne for a longer time, may go undetected via traditional detection methods, and can reach the bronchi, inducing bronchial constriction [52], especially if physical exercise and/or oral respiration are performed. The priming effect is defined as an increase in the reactivity of the mucous membranes following repeated exposures to pollen, leading to symptoms that may occur paradoxically on days with lower concentrations of pollen grains. This phenomenon has been demonstrated formerly for nasal symptoms [53], although subsequent studies with both experimental and natural exposure have inconsistent results [54].

It is important that patients and their families know the symptoms of both asthma and allergic rhinoconjunctivitis so that they can be detected and treated as early as possible. Each patient with pollinosis should have an action plan established by a physician, which includes a daily (preventive) treatment and “rescue” treatments to be used on demand in case of the appearance of symptoms. Apart from pharmacological treatments, the only measures that can slow down the evolution of a seasonal allergy are allergen avoidance and specific immunotherapy. Although it is practically impossible for patients to avoid total contact with allergens coming from pollen, some general measures should be considered (Table 4), the main one being to know which pollen produces their symptoms as well as the pollination period of the species in their area of residence, information that is available on the Internet or through specific mobile apps. FFP2 self-filtering masks should be used, which retain 95% of airborne particles larger than 0.1 µm. On the other hand, there are intranasal filters, with pores of 1 µm. Surgical masks are not suitable for filtering environmental pollen.

Sensitization to pollen allergens that occurs in early childhood may remain unnoticed, and most individuals allergic to pollen begin to have symptoms at 6–7 years of age. In at-risk preschoolers, with specific IgEs positive to pollens and/or allergic parents, Matricardi has suggested that immunological intervention by means of specific AIT in a preventive manner, before the onset of rhinoconjunctivitis and/or asthma, could induce immunotolerance [18], taking advantage of an immunological window of opportunity at these ages, this measure being more effective than mere allergen avoidance.

### 11.2. Molds

It is estimated that more than 1 million fungal species exist, although only 80,000 have been described, of which 112 genera are thought to contain allergens [55]. There are four genera associated with the development of respiratory allergy in humans: *Alternaria*, *Cladosporium*, *Penicillium*, and *Aspergillus*. Fungi can behave as saprophytes, symbiotics, or parasites. They live on decaying organic matter, soils, textiles, food, and plants, and require an optimum temperature of 25 °C (4–40 °C) for their development.

Molds play an important role in the development of respiratory allergy, as they are associated with rhinitis, asthma, particularly severe asthma [56], ER visits, hospitalizations, and ICU admissions. The genera *Alternaria* and *Cladosporium* are the most frequently associated with allergic respiratory diseases, with *Alternaria* being the one with the highest prevalence of sensitization. Despite Alt a1 being the *Alternaria alternata* major allergen, other allergens, such as Alt a 6 or Alt a 14, have been suggested to be included in the diagnosis panel of fungal allergy [57]. The prevalence of sensitization to molds in the general population ranges between 3 and 10%, rising to 10–20% in patients with asthma and/or rhinitis [55], being more frequent in childhood. In addition, by stimulating the innate immune system, they can enhance the inflammation caused by other unrelated allergens, such as pollen or mites; polysensitization is frequent.

Mycelia and fungal spores contain allergenic material, acting both as indoor allergens and, since light promotes sporulation (asexual reproduction), outdoor allergens, predominantly in agricultural regions. Fungal spores show a broad spectrum of different shapes and sizes, ranging from less than 2 to 250 μm, and constitute the largest component of all environmental aerobiological particles in our environment [58], exceeding the concentration of pollen grains. Spore concentration is strongly influenced by climatic factors (temperature, rainfall, winds, and relative humidity) and circadian patterns (light and dark). The highest levels in the Northern Hemisphere are found between June and October, when nutritional sources are available, and they decrease in winter. As with pollens, spore concentration does not fully correlate with human exposure, because there are fragmented spores and submicron particles unaccounted for in the samplers that contain appreciable amounts of allergens capable of depositing in the respiratory tract, producing asthma. Spore concentrations of Alternaria greater than 100 spores/m^3^ are believed to induce allergic symptoms, whereas the equivalent value for Cladosporium is estimated to be 3000 spores/m^3^ [55]. A meta-analysis [59] found an increased risk of respiratory allergy in children exposed at an early age to fungi/dampness, which should be considered when establishing primary prevention measures for respiratory problems associated with indoor allergens.

## 12. Relationship between Environmental Factors and Respiratory Allergic Diseases

The interaction between genetic predisposition and external factors programs immune function early in life, after moving from a protective intrauterine environment to external exposure through the skin, respiratory system, and digestive tract [60]. During the last months of pregnancy and the first months of extrauterine life, immunological maturation takes place, producing the first responses to allergens presented to the fetus through the placenta. Depending on a series of events, the child may be marked for life with an adequate Th2/Th1 balance, or with a Th2 predominance typical of atopic patients, which will favor the appearance of allergy problems later.

The increase in the prevalence of allergic diseases in Western-lifestyle countries has been described as a real “epidemic”. The causes of this increase are not fully known, and many hypotheses have been put forward to explain this phenomenon, most related to changes in lifestyle and eating habits as well as environmental and family factors, such as the reduction in typical childhood infections, sedentary lifestyles, the consumption of ultraprocessed foods, obesity, exposure to tobacco smoke, antibiotic abuse, and increased time spent indoors, among other factors. There are complex interactions between air pollution and climate change on the one hand, and allergens and adjuvants on the other, and the immune system that they influence, involving physical, chemical, and biological factors [61].

### 12.1. Air Pollution

Two patterns of air pollution have been described: Type I, typical of developing countries, in which SO_2_ and dust predominate, and Type 2, present in developed countries, derived from fossil fuels, and characterized by organic compounds, ozone, and fine particles, generated by industry and automobile traffic, the latter one being associated with an increase in the prevalence of asthma [62] in both atopic and nonatopic populations. Pollutant particles include inorganic components (sulfates, nitrates, ammonium, chloride, and trace metals), elemental and organic carbon, biological components (bacteria, spores, and pollens), and adsorbed organic compounds [63]. Those particles, defined as PM10 and PM2.5 depending on whether their diameters are less than 10 and 2.5 μm, respectively, when mixed with atmospheric gases (ozone, sulfur nitric oxides, and carbon monoxide) can generate environmental aerosols. Although the underlying mechanisms are incompletely known, pollution can produce direct epithelial damage and increase oxidative stress as well as inflammation in airways [64].

Diesel exhaust particles (DEPs) are a major source of especially ultrafine particles (UFPs), with diameters smaller than 100 nm, that show a high alveolar deposition fraction and the ability to enter the circulation and induce inflammation, resulting in systemic effects. DEPs include polyaromatic hydrocarbons (PAHs) that have very important biological effects, such as increasing Th2 and Th17 adaptive immune responses, as seen in allergy and asthma, and the dysregulation of antiviral immune responses [65,66]. Moreover, DEPs seem to prime cells to react more rapidly to native pollen exposure, especially inflammation-related genes, a factor known to facilitate the development of allergic sensitization [67].

Pollutants interact with pollens and molds, leading to quantitative and qualitative changes in their allergens: they agglomerate with PAH, increase the release of allergens from pollen grains and fungal spores, facilitate their transport into the airways, and act as adjuvants by enhancing allergen expression and stimulating IgE-mediated responses [68]. Pollen grains are considered not only carriers of allergens, but also of pollutants, which contributes to the fact that the prevalence of pollinosis is twice as high in urban areas as in rural areas, even though pollen concentrations are much higher in the latter [69]. The first description of the influence of diesel engine fumes on the increased prevalence of pollinosis was made by Dr. Muranaka et al. in 1986, after observing in a murine model how the IgE antibody response to Japanese cedar pollen increased significantly when exposed to pollens mixed with DEPs [70]. Diaz Sanchez et al. performed a nasal challenge with ragweed pollen mixed with diesel pollutants in allergic patients. They found that the increase in specific IgEs present in nasal secretion was twenty times greater than the increase produced after a nasal challenge with nondiesel ragweed pollen [71]. There are several mechanisms by which diesel can enhance the allergic response and thus the symptoms of rhinitis and asthma:

- Binding aeroallergens, which allows their higher concentration and permanence in the air.
- Decreasing mucociliary clearance and increasing the permeability of the respiratory epithelium to allergens, which thus remain longer in the respiratory mucosa and therefore have greater exposure to the immune system.
- Increasing the allergenicity of pollen and the production of chemical mediators responsible for allergic reactions and airway inflammation.

There is substantial evidence for the separate effects of outdoor pollutants and allergens on asthma and respiratory allergy from numerous small experimental investigations; however, two systematic reviews failed to find that pollution, heat, and airborne allergens have a synergistic effect [72,73]. This lack of a correlation between the evidence from epidemiological studies versus that from experimental studies can be explained by different causes: (1) pollen and spore traps may be far away from the pollution meters, (2) levels of pollen grains measured in the samplers may not represent the actual exposure of individuals to their allergens, either because the traps are not at ground level or because submicron particles go unnoticed, (3) it does not take into account the priming effect, (4) the epidemiological studies measure daily averages, but what constitutes a risk factor are the peak levels, and (5) the heterogeneity between types of allergen source (grass, trees, and molds) and types of pollution particles (NO_2_, ozone, SO_2_, CO, and PM10), among other reasons. Epidemiological studies that attempt to verify whether there is a synergistic effect between outdoor allergen sources and outdoor pollutants are methodologically complex and must control numerous variables to generate quality evidence [73].

With respect to indoor allergens, it has been observed that the levels of IL-33 and its receptor, ST2, are much more markedly elevated in bronchoalveolar lavage and lung tissue after exposure to the combination of DEP + HDM compared to exposure to these elements separately [74].

### 12.2. Climate and Meteorology

Human activity, with the use of intensive crops, fertilizers, and transgenic species, is responsible for the increased production of pollen allergen components and their release into the atmosphere. For years, temperatures have been rising worldwide, and this warming affects the physiology and distribution of plants and fungi, causing pollination periods to start earlier and persist longer as well as more intensely, in addition to increasing spore production and modifying the allergenicity of fungi [61,75]. It is also observed that different plant species are progressively spreading their habitats to areas where they were not present before. An example of this is the ragweed, native to Eastern Europe, for which it is predicted that in the coming decades we will see a significant increase in the frequency of sensitization in Western European populations [19,76].

Rapid atmospheric changes, such storms, can increase the levels of pollens as well as fungal spores and disperse them further. So-called “thunderstorm asthma” occurs during the pollen (or mold) season and is characterized by the sudden onset and severity of asthma attacks due to submicron allergenic particles, usually contained within pollen grains and spores that are released after an osmotic rupture, reaching a size of less than 5 μm, allowing them access to the bronchial airways [75]. Idrose et al., in a systematic review, conclude that the combination of high pollen levels and storm generation does not always result in increased consultations and hospitalizations for asthma attacks, suggesting that fungal or other environmental factors may be important and therefore merit further investigation [77].

## 13. Allergen Avoidance in Allergic Respiratory Diseases

The route of exposure and the amount of allergens determine the risk of allergic sensitization. Thus, exposure by the digestive route tends to generate tolerance mechanisms more easily, whereas the transdermal or respiratory route involve a greater risk of sensitization [78].

Allergen avoidance is a preventive strategy at three levels:

- Primary, in at-risk patients (with a family or personal history of atopy).
- Secondary, in sensitized patients who have not yet presented associated symptoms (or these are mild).
- Tertiary, in allergic patients, to reduce the risk of symptom exacerbations.

Avoidance measures may fail due to a perceived lack of efficacy in symptom reduction, or the lack of time needed to achieve sufficient allergen reduction. The efficacy of different methods is difficult to establish, and most have low levels of evidence [28]. The effect of interventions is limited, and meta-analyses may show partial beneficial results in some parameters (i.e., nasal symptoms and quality of life), but not in others (i.e., lung function and bronchial symptoms). Such interventions should be included within a series of general measures grouped in health education, such as knowledge of their own disease, exacerbation triggers, symptom recognition, self-management, and improved adherence to treatment as well as inhaled medication administration technique, among others.

## 14. Treatment of Allergic Respiratory Diseases

The therapeutic arsenal for allergic asthma and rhinoconjunctivitis includes the following measures:

- Symptomatic treatments: Antihistamines (oral or topical on nasal or conjunctival mucosa) and inhaled bronchodilators. They relieve symptoms temporarily, so they are used as on-demand medication.
- Anti-inflammatory treatments: Leukotriene receptor antagonists (oral) and glucocorticoids (nasal and inhaled) used preventively on a perennial or seasonal basis, depending on the type of respiratory allergy. Systemic corticosteroids are reserved for the treatment of asthma attacks or in the maintenance of severe asthma not controlled with other treatments.
- Biological treatments: Directed against cytokines or IgEs, which act as therapeutic targets in the different subtypes (endotypes or phenotypes) of asthma. As they are directed against specific targets, they are not able to control all of the clinical and functional aspects that characterize asthma, nor eliminate the risk of future exacerbations. Currently, none of the biologicals approved for the treatment of severe asthma demonstrated any disease-modifying effect [79], so this type of treatment is actually effective as long as it is administered, but no permanent effect has been demonstrated after the treatment is stopped.
- Immunotherapy: With the drugs of the previous sections used in monotherapy or in combination, some intermediate targets (biological drugs) or the consequences (anti-inflammatory and symptomatic treatments) of respiratory allergy would be partially treated, but not the etiology (inflammation of allergic cause). In cases of respiratory allergy, pharmacological and biological treatments should be combined with allergen-specific immunotherapy, consisting of the administration of repeated doses of allergenic extracts for 3 to 5 years, to induce prolonged immunological tolerance to responsible allergens. AIT is currently the only treatment capable of altering the natural history of allergic diseases [80], preventing the development of asthma in patients with rhinoconjunctivitis [81] and the acquisition of new sensitizations in monosensitized patients [18]. Since it has been shown that most subjects with allergic sensitization before the age of 8 will develop rhinitis and/or asthma before adulthood [82], those immunological changes occurring at this window of opportunity could lead to tolerance to the allergen, which would be accompanied by a reduction in symptoms and the need for medication as well as other health resources, as well as an increase in the quality of life perceived by patients. In a review of five meta-analyses, AIT proved to be at least as effective as pharmacotherapy in controlling the symptoms of seasonal allergic rhinitis as early as in the first season of treatment [83].
- General measures acting on modifiable factors, such as tobacco smoke exposure, pollution, global warming, and lifestyle changes.

## Figures and Tables

**Table 1:** Main characteristics of respiratory allergens.

Acidic extracellular proteins or glycoproteins that induce an IgE response. Highly hydrophilic.
Not intrinsically toxic substances.
Molecular size of less than 100 kDa (mainly between 3 and 60 kDa).
They are usually harmless proteins in the absence of an allergic reaction.
They are usually resistant to the actions of heat, pH, and enzymes.
Some with proteolytic enzymatic activity, especially mites and fungi.
Several isoforms with variable allergenicity.
Induction of T cell proliferation.
Intra- and interspecific cross-reactivity.
Epitopes: polypeptide sequences of 8 to 15 aa. Their number is highly variable.
According to the frequency of recognition:
o Major allergens (>50% of sensitized individuals).
o Minor allergens (<50% of sensitized individuals).

**Table 2:** Thommen’s postulates. Requirements for a substance to be considered a respiratory allergen.

To be present in the environment.
2. In sufficient quantities to induce sensitization.
3. Allergic sensitization is demonstrable by a skin test or by a specific IgE.
4. Produce symptoms after inhalation.

**Table 3:** Effectiveness of avoidance measures for pets. Table adapted from [41]. Evidence level graded according to the 2011 Centre for Evidence-Based Medicine (Oxford) system [42].

Measure	Level of Evidence	Grade of Recommendation
Thermostatically controlled laminar flow	2	B
Washing pets ≥ two times per week	4	C
Removing pet from the bedroom	5	D
Bed covers and pillowcases/wash linen with chemicals	5	D
Air purifiers and vacuum cleaners with HEPA filters	5	D
Binder lotions that encapsulate allergens on the fur of the animal	5	D

**Table 4:** Instructions to avoid pollen exposure.

In the months of spring (summer in some regions) it is not advisable to go to the countryside, parks, gardens, or places with abundant vegetation.
2. Areas with grass should be avoided, especially if it is freshly cut.
3. In the less hot hours of the day (dusk and dawn) the air contains more pollen in suspension.
4. Keep bedroom windows or balconies closed as long as possible, especially at night, when the concentration of atmospheric pollen increases.
5. It may be useful to spray water towards the top of the room to carry pollen to the floor, and to protect windows with a dense mesh (mosquito net), which should be cleaned daily with a damp cloth.
6. Outside, sunglasses, useful in cases of seasonal conjunctivitis, and approved antipollen masks can be used.
7. If traveling by car, keep the windows closed. Check that the antipollen filters are in good condition.
8. Clean the house without shaking with a vacuum cleaner or damp cloth.
9. Consult (press, television, the Internet, and applications for smartphones) the data on pollination. Take precautions when there are more than 50 grains of pollen per cubic meter of air, because from that amount symptoms may occur.
10. Start the preventive medication indicated by your physician and know the symptomatic treatment to be used if necessary.

## Data Availability

Not applicable.
